# Effectiveness of remineralizing dentifrices against caries lesions: A systematic approach

**DOI:** 10.1016/j.jtumed.2025.11.007

**Published:** 2025-12-16

**Authors:** Fatima T. Zahra, Mehmood Asghar, Muhammad S. Zafar, Asma T. Shah, Muhammad Kaleem

**Affiliations:** a Basic Dental Sciences, Army Medical College, National University Medical Science, Rawalpindi, Pakistan; b Department of Clinical Sciences, Ajman University, Ajman, AE, United Arab Emirates; c Center of Medical and Bioallied Health Sciences Research, Ajman University, Ajman, United Arab Emirates; d School of Dentistry, University of Jordan, Amman, Jordan; e Interdisciplinary Research Centre in Biomedical Materials (IRCBM), COMSATS University Islamabad, Lahore Campus, Lahore, Pakistan

**Keywords:** Bioactive glass, Casein phosphopeptide-amorphous calcium phosphate (CPP-ACP), Dentifrice, Dentine caries prevention, Fluoride, Remineralization, إعادة التمعدن, الزجاج الحيوي, الفسفوببتيد الكازئيني- فوسفات الكالسيوم اللامتبلور, الفلوريد, الوقاية من نخر العاج, معاجين الأسنان

## Abstract

**Objectives:**

Dental caries remains a prevalent global oral health problem. Fluoride promotes enamel remineralization but is less effective on deeper lesions. Non-invasive agents, including bioactive glass and casein phosphopeptide-amorphous calcium phosphate (CPP-ACP), aim to enhance remineralization and prevent caries.

**Methods:**

A systematic review of in vitro studies was conducted following PRISMA 2020 guidelines. PubMed, Ovid, Cochrane Library, ScienceDirect, and Scopus were searched for studies published before 31 December 31, 2024. Titles, abstracts, and full texts were screened independently by two reviewers. Risk of bias was assessed using a modified CONSORT checklist.

**Results:**

Among 142 records identified, 38 were screened, 21 full texts retrieved, and 11 studies met the inclusion criteria. Bioactive glass-based dentifrices generally outperformed fluoride-only formulations, with greater mineral deposition, improved surface microhardness, and reduced lesion depth. The assessment methods included surface microhardness (Vickers or Knoop hardness testing), transverse microradiography, and scanning electron microscopy.

**Conclusions:**

Bioactive glass and CPP-ACP exhibited promise for in vitro remineralization, and bioactive glass was consistently superior to fluoride alone. Methodological variability and limited randomization, blinding, and sample size justification reduced the reproducibility of studies. Well-designed in situ and clinical studies are needed to confirm clinical applicability.

## الملخص

**أهداف البحث:**

تُعدّ نخور الأسنان أحد أكثر مشكلات صحة الفم شيوعًا عالميًا. يعمل الفلوريد على تعزيز إعادة تمعدن الميناء، إلا أن فاعليته تقلّ في الآفات العميقة. تهدف العوامل غير الجراحية، مثل الزجاج الحيوي والفسفوببتيد الكازئيني- فوسفات الكالسيوم اللامتبلور، إلى تعزيز إعادة التمعدن والوقاية من النخر السني.

**طرق البحث:**

أُجري استعراضٌ منهجيٌّ للدراسات المخبرية وفقًا لإرشادات بيان "بريزما" لعام 2020م. تم البحث في قواعد البيانات: ببمد، أوفيد، مكتبة كوكران، ساينس دايركت، وسكوبس عن الدراسات المنشورة حتى 31 ديسمبر 2024م. جرت مراجعة العناوين والملخصات والنصوص الكاملة بشكلٍ مستقل من قبل مقيِّمَين اثنين. وقد تم تقييم خطر التحيّز باستخدام قائمة تحقق معدّلة من مقياس "كونسورت".

**النتائج:**

من أصل 142 سجلًّا تم تحديده، جرى فحص 38، واسترجاع 21 نصًّا كاملاً، واستوفت 11 دراسة معايير الإدراج. أظهرت معاجين الأسنان المحتوية على الزجاج الحيوي أداءً أفضل من تلك المحتوية على الفلوريد فقط، من حيث زيادة ترسّب المعادن وتحسين صلادة السطح المجهري وتقليل عمق الآفة. شملت طرائق التقييم قياس الصلادة الميكروية السطحية، والتصوير المقطعي الإشعاعي المجهري العرضي، والمجهر الإلكتروني الماسح.

**الاستنتاجات:**

أظهر كلٌّ من الزجاج الحيوي والفسفوببتيد الكازئيني- فوسفات الكالسيوم اللامتبلور فاعلية واعدة في إعادة التمعدن داخل المختبر، وكان الزجاج الحيوي متفوقًا بانتظام على الفلوريد وحده. إلا أن التباين المنهجي، وضعف العشوائية والتعمية وتبرير حجم العينة، قلّل من إمكانية تكرار النتائج. لذا، هناك حاجة إلى دراساتٍ مصمَّمةٍ جيدًا تُجرى على نماذج فموية حية ودراساتٍ سريرية لتأكيد إمكانية تطبيق النتائج في الممارسة الإكلينيكية.

## Introduction

Dental caries remains one of the most prevalent oral health problems worldwide, affecting individuals in all age groups.^1^, ^2^, ^3^ The progression of caries involves an imbalance between demineralization and remineralization, leading to loss of minerals from the enamel and dentine surface due to acid production by cariogenic bacteria..^4^ Traditional fluoride-based intervention has long been used to enhance enamel remineralization and reduce lesion progression.^5^^,^^6^ However, fluoride alone has a limited effect on restoring the lost mineral content in deeper lesions, thereby prompting research into alternative bioactive remineralization strategies.^6^^,^^7^ In particular, bioactive materials such as bioactive glass, casein phosphopeptide-amorphous calcium phosphate (CPP-ACP), and metal fluoride-modified formulations have emerged as promising agents for non-invasive caries management.^8^, ^9^, ^10^

Bioactive glass formulations consisting of calcium sodium phosphosilicate (e.g., Novamin) and fluoride-doped variants (e.g., BiominF) release calcium, phosphate, and fluoride ions to stimulate hydroxyapatite formation and remineralization.^11^, ^12^, ^13^ Similarly, CPP-ACP has been investigated for preventing demineralization and promoting enamel repair due to its calcium- and phosphate-stabilizing properties.^8^ Recent studies also suggest that incorporating these bioactive glass materials into dentifrices and preventive treatments can dramatically improve their ability to restore lost mineral contents and prevent lesion progression.^14^, ^15^, ^16^, ^17^, ^18^, ^19^, ^20^ The large amount of clinical evidence shows the promise of these approaches but methodological inconsistencies in in-vitro studies prevent the definition of clear clinical guidelines.^15^

Much of the research into remineralization is complicated by the wide diversity of protocols employed, with differences in the pH cycling methods, durations of demineralization and remineralization, and outcome measures.^16^ Moreover, many studies failed to apply critical methodological requirements such as randomization, blinding, and sample size justification, which are all essential for improving the validity and reproducibility of findings,^21^ making it difficult to determine the most effective remineralization strategies.^22^ Thus, it is necessary to thoroughly review in vitro studies and organize the data produced in an appropriate manner to obtain a better understanding of the advantages and disadvantages of different remineralization methods and their future implications.

Fluoride has been shown to influence enamel repair kinetics, with a threshold concentration above which remineralization ceases, highlighting the limitations of in vitro systems due to diffusion, crystal growth, and ion gradients.^23^ Considerable variability exists in terms of fluoride release and cariostatic efficacy among toothpastes and restorative materials,^24^^,^^25^ and clinical factors such as dentifrice use and diet further impact outcomes.^26^ In addition to fluoride, bioactive materials including bioactive glass, glass–ceramics, and calcium phosphate composites exhibit potential for enhancing enamel remineralization, although material-dependent differences persist.^27^, ^28^, ^29^ These findings highlight the need to specify standardized in vitro and clinical protocols for reliably assessing the remineralization potential and comparing similar results.

**Figure 1:**
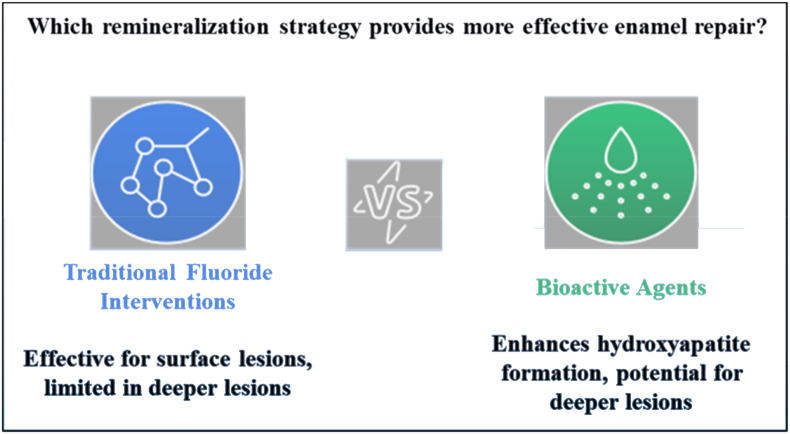
Comparison of remineralization strategies for effective enamel repair.

In the present study, a modified CONSORT checklist was used to systematically review the quality and methodological strength of in vitro studies of bioactive remineralization agents.^30^ By evaluating factors such as study design, intervention protocols, outcome measures, and statistical analysis, this review provides valuable insights into the reliability of current evidence, ultimately contributing to the development of more standardized and clinically relevant remineralization strategies, as depicted in Figure 1.

## Methodology

### Study objective and inclusion parameters

To assess the effectiveness of bioactive glass-containing dentifrices, metal fluoride formulations, and CPP-ACP at enhancing dentine remineralization and preventing caries progression based on in vitro evidence, a systematic review was performed following the updated guidelines of the Preferred Reporting Items for Systematic Review and Meta-Analysis (PRISMA 2009). The following PICO framework was designed to address the research objective:

• P (Population): In vitro studies of dentine remineralization
• I (Intervention): Bioactive glass-containing dentifrices, metal fluoride formulations, and CPP-ACP
• C (Comparison): Untreated control groups
• O (Outcome): Mineral deposition, surface microhardness, lesion depth reduction, and overall remineralization effectiveness

### Inclusion criteria

The reviewed articles were filtered using the following inclusion criteria to meet the aims of the reviewer.

1. All methodological approaches were deemed eligible.
2. The study used human or animal source (bovine) teeth with natural or artificially induced caries.
3. Studies that measured the remineralization efficacy of bioactive glass either alone or in combination with other agents.
4. Outcome measure: All studies related to remineralization, caries prevention, and safety.
5. Full-text articles available.
6. Written in English.

### Exclusion criteria

1. Studies published in languages other than English were excluded.
2. Studies were excluded if the full text was not available.
3. Articles not focused on dentine caries
4. Animal studies, case reports, and case series.
5. Systematic review and grey literature

### Literature search and selection

Article selection was conducted by two researchers. Five online databases were searched electronically for relevant literature. MEDLINE, PubMed, Ovid, Cochrane Library, and Scopus were searched to identify relevant studies published up to December 31, 2024 (Table 1). Medical subject headings (MeSH) and Boolean operators were selected to retrieve the articles in the database search terms. A different set of keyword search criteria was used for different forms of efficient searching on web pages. In total, 142 records were identified across the included databases (Cochrane Library: 13; Ovid: 4; PubMed: 34; ScienceDirect: 15; Scopus: 87). After sequential screening of titles/abstracts (n = 38) and full texts (n = 21), 11 studies remained eligible for inclusion, as summarized in Table 2.

**Table 1:** Databases used and MeSH strategies.

Database	Search Query
PubMed	(((Fluoride ion OR fluorine compound OR sodium fluoride (NaF) OR stannous fluoride (SnF2) OR monofluorophosphate (MFP) OR fluoride treatment OR fluoridation) AND (bioactive ceramics OR bioglass OR silicate glass OR calcium phosphate glass OR osteoinductive glass OR glass ionomer)) AND (dentifrice OR toothpaste OR fluoride gels)) AND ((dentifrice OR toothpaste OR fluoride gel) AND (caries OR demineralization OR cavity))
Ovid	(Fluoride ion OR fluoride treatment) AND (caries OR demineralization)
ScienceDirect	(Fluoride ion OR fluoride treatment) AND (bioactive glass OR remineralization) AND (dentifrice OR toothpaste) AND (caries OR demineralization)
Cochrane library	(Fluoride ion OR fluoride treatment) AND (bioactive glass OR remineralization) AND (dentifrice OR toothpaste) AND (caries OR demineralization)
Scopus	(((Fluoride ion OR fluorine compound OR sodium fluoride (NaF) OR stannous fluoride (SnF2) OR monofluorophosphate (MFP) OR fluoride treatment OR fluoridation) AND (bioactive ceramics OR bio glass OR silicate glass OR calcium phosphate glass OR osteoinductive glass OR glass ionomer)) AND (dentifrice OR toothpaste OR fluoride gel)) AND ((dentifrice OR toothpaste OR fluoride gel) AND (caries OR demineralization OR cavity))

**Table 2:** Database search and screening summary.

Step/Database	Number of References
Total references retrieved	142
Cochrane library	13
Ovid	4
PubMed	34
ScienceDirect	15
Scopus	87
Duplicates removed	0
Title and abstract screened	38
Full-text screened	21
Studies excluded	10
Studies included	11

Only articles published by December 31, 2024, were included, with no search limitations. The articles were stored using EndNote (Clarivate Analytics, EndNote 20, 2021). After removing duplicates, initial article screening was performed based on titles and abstracts. Full texts of screened articles were retrieved and then screened according to PRISMA guidelines.^31^ Full-text screened articles were also searched manually for references to include eligible studies.

**Figure 2:**
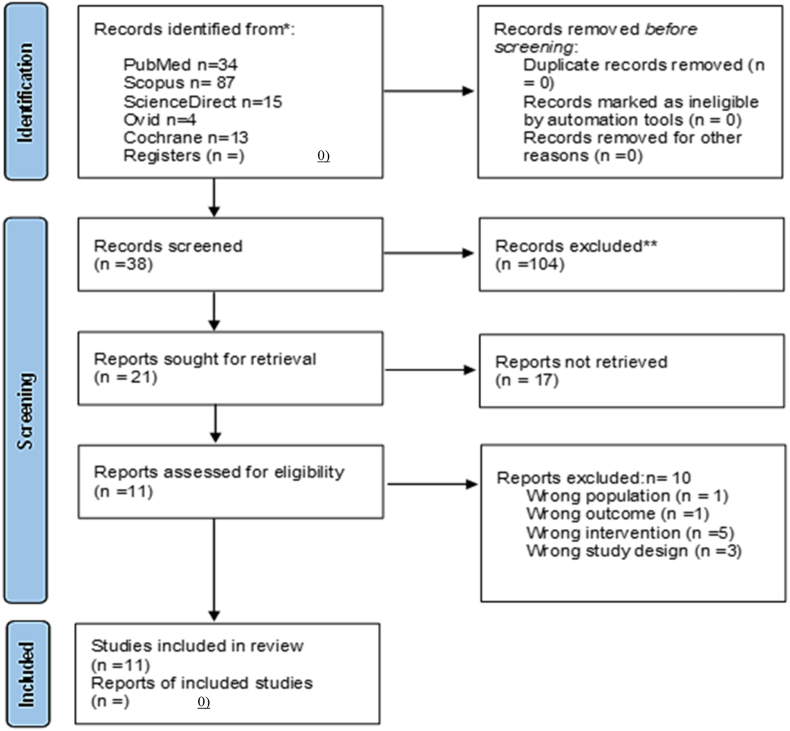
Systematic review process based on the updated PRISMA 2020 guidelines.

The process based on selecting, screening, including, and excluding studies is depicted in the PRISMA 2020 flowchart (Figure 2).^31^

### Data collection and extracted variables

To ensure unbiased selection, reviewers FTZ and MA independently screened the titles and abstracts against the inclusion criteria. Any disagreements were resolved by a third referee (MK). To ensure eligibility, the full texts of short-listed studies was retrieved and critically assessed before final inclusion in the review process.

### Assessment of risk of bias in studies included

The data from selected studies were extracted using a customized Excel spreadsheet. Two independent reviewers (FTZ and MA) assessed the risk of bias and the level of evidence in the selected studies using the modified Consolidated Standards of Reporting Trials (CONSORT) checklist^32^ for reporting in vitro studies of dental materials. Studies that were excluded based on the inclusion criteria are listed in Table 3.

**Table 3:** Studies not eligible for systematic review.

Source Author and Year	Country	Study Aims and Rationale	Reason for Ineligibility
Christoffersen, Christoffersen^23^	Denmark, Netherlands	Investigated the effect of fluoride ions on remineralization of demineralized enamel	Not focused on dental caries
Chung, millett^24^	UK	Studied fluoride release and cariostatic ability of orthodontic bonding materials	Focus on orthodontic bonding not general dental caries
De Queiroz, Nouer^26^	Brazil	In vivo evaluation of effect of fluoride dentifrice on demineralization/remineralization at the bracket/enamel interface	Orthodontic study not focused on general dental caries
Eggert and Neubert^25^	Germany	In vitro investigation of fluoride ion release from toothpaste compounds	Focus on fluoride toothpaste rather than dental caries management
Milly, festy^27^	UK	Examined bioactive glass in white spot lesion remineralization	Study on white spot lesions rather than general dental caries
Qiu, Lu^28^	China	Evaluated a novel bioactive glass–ceramic for dental applications	Focus on bioactive glass development rather than dental caries treatment
Degli Esposti, Zheng^29^	–	Investigated composite materials made of amorphous calcium phosphate and bioactive glass nanoparticles for preventive dentistry	Not directly related to dental caries treatment
Chung, millett^24^	UK	Analyzed fluoride uptake and release in orthodontic materials	Focus on orthodontics not general caries management
Eggert and Neubert^25^	Germany	Investigated fluoride ion release in a permeation model	Not focused on dental caries treatment
Qiu, Lu^28^	China	Evaluated remineralization potential of novel bioactive glass materials	Not specific to dental caries treatment

**Table 4:** CONSORT-based quality assessment of in vitro studies.

Study (Author, Year)	Title & Abstract	Introduction	Objectives	Sample Size Justification	Randomization & Blinding	Outcome Measures & Statistical Analysis	Overall Quality
Bakry, abbassy^11^	Yes	Yes	Yes	No	No	ANOVA, microhardness, SEM	Moderate
Dai, mei^12^	Yes	Yes	Yes	No	No	Micro-Computed Tomography (CT), mineral analysis, ANOVA	Moderate
de moura, de melo simplício^34^	Yes	Yes	Yes	No	No	Microhardness, SEM, ANOVA	Moderate
Parkinson, siddiqi^16^	Yes	Yes	Yes	No	No	Surface hardness, TMR, ANOVA	Moderate
Suryani, Gehlot^14^	Yes	Yes	Yes	No	No	Microhardness, SEM, ANOVA	Moderate
Wang, mei^8^	Yes	Yes	Yes	No	No	Knoop hardness, SEM, Tukey's test	Moderate
Ali, farooq^21^	Yes	Yes	Yes	No	No	Vickers hardness, statistical analysis reported	Moderate
Dai, mei^12^	Yes	Yes	Yes	No	No	Micro-CT, lesion depth analysis	Moderate
Khamverdi, farmani^15^	Yes	Yes	Yes	No	No	Microhardness, SEM, ANOVA	Moderate
Suryani, Gehlot^14^	Yes	Yes	Yes	No	No	Vickers hardness, SEM, ANOVA	Moderate
Aras, Celenk^33^	Yes	Yes	Yes	No	No	Microhardness, SEM, ANOVA	Moderate

CT, Computed Tomography.

These studies were excluded based on the predefined criteria, including language, type of study, lack of focus on dental caries, and non-human studies.

## Results

### Key features of studies included

The initial search yielded a total of 142 papers from electronic databases. Using EndNote 20 software, the studies were then screened (titles and abstracts) to obtain a total of 38 studies. Full texts were retrieved for 21 of these studies for detailed analysis of eligibility for inclusion in the systematic review, and one study was not included because the full text could not be retrieved. Subsequently, 10 studies were excluded for various reasons, and 11 studies were included in the final analysis. The general characteristics of studies included in the systematic review are shown in Table 5, and the data from previous studies and significant findings are listed in Table 6. Heterogeneity among the studies included in the review prevented meta-analysis.

**Table 5:** General characteristics of studies included.

Author and Year	Country	Study Design	Remineralization Measurement Device	Time points
Ahmed samir bakry et al. (2020)	Not specified	In vitro	SEM, EDS, TMR, microhardness testing	4 days
Lin Lu Dai et al. (2022)	China	In vitro	Micro-CT, mineral loss analysis	4 days
Marcoeli silva de moura et al. (2019)	Brazil	In vivo	Microhardness testing, SEM	28 days
Parkinson et al. (2017)	USA	In situ	Surface microhardness, TMR, SEM	28 days
Suryani et al. (2021)	India	Ex vivo	Vickers microhardness, SEM	15 cycles
Yu Wang et al. (2016)	China, New Zealand	In vitro	Knoop microhardness, SEM, AFM	15 days
S. Ali et al. (2019)	Not specified	In vitro	Vickers microhardness testing	24 h
Dai LL et al. (2022)	China	In vitro	Micro-CT, lesion depth analysis	Not specified
Zahra Khamverdi et al. (2023)	Iran	In vitro	Microhardness testing, SEM	14 days
Paras mull Gehlot et al. (2021)	India	Ex vivo	Vickers microhardness, SEM	28 days
Aras et al. (2019)	Not specified	In vitro	SEM, microhardness testing	Not specified

**Table 6:** Summary of results from studies included.

Study (Author, Year)	Sample Type	Substrate	Caries Type	Measured Outcomes & Equipment	Reported Findings
Bakry, abbassy^11^	In vitro	Enamel	White spot lesions	TMR, SEM, EDS, microhardness	BiominF® paste reduced mineral loss
Dai, mei^12^	In vitro	Dentine	Artificial lesions	Micro-CT, mineral loss analysis	Strontium-doped bioactive glass improved mineralization
de moura, de melo simplício^34^	In vivo	Enamel	Orthodontic lesions	Microhardness, SEM	Fluoridated dentifrice was most effective
Parkinson, siddiqi^16^	In situ	Enamel	Artificial lesions	Surface microhardness, TMR, SEM	CSPS did not enhance effect of fluoride
Suryani, Gehlot^14^	Ex vivo	Enamel	Erosion lesions	Vickers microhardness, SEM	bioactive glass (BAG) and CPP-ACPF obtained highest remineralization
Wang, mei^8^	In vitro	Enamel	Early caries lesions	Knoop hardness, SEM, atomic force microscopy (AFM)	NovaMin and Pro-Relief were highly effective
Ali, farooq^21^	In vitro	Enamel	Artificial lesions	Vickers microhardness	BiominF® was more effective than Novamin®
Dai, mei^12^	In vitro	Dentine	Root caries	Micro-CT, lesion depth analysis	Strontium-doped bioactive glass reduced lesion depth
Khamverdi, farmani^15^	In vitro	Enamel	Artificial lesions	Microhardness, SEM	Nano-bioactive glass improved remineralization
Suryani, Gehlot^14^	Ex vivo	Enamel	Erosion lesions	Vickers microhardness, SEM	BAG and CPP-ACPF were most effective
Aras, Celenk^33^	In vitro	Enamel	Artificial lesions	SEM, microhardness testing	BAG exhibited significant remineralization potential

AFM, atomic force microscopy; BAG, bioactive glass.

### Assessment of potential bias in selected studies

According to the modified CONSORT checklist (Table 4), the 11 studies included were characterized by moderate quality with a low risk of bias, clear objectives, appropriate methodologies, and valid statistical analyses. However, none of the studies incorporated randomization, blinding, or sample size justification, which are critical for minimizing bias. The modified CONSORT checklist (Table 4) highlights these methodological strengths and limitations, emphasizing the need for standardized research protocols in in-vitro remineralization studies.^30^

## Summary of results

### Overview of studies included

In the following, the findings are synthesized from the 11 studies included in the systematic review with respect to the substrate type, caries nature, measured outcomes, specimen dimensions, group size, and grouping of intervention or restoration. The effectiveness of fluoride-based bioactive glass dentifrice at preventing dentine caries was the same across all of these factors.

### Sample type and substrate

Most of the studies utilized human enamel and dentine specimens, although some employed bovine enamel models due to their similar structure to human teeth.^8^ Human enamel is considered the gold standard for remineralization research, but bovine models are often used as they are readily available and provide greater standardization. However, differences in mineral density and permeability between human and bovine enamel may influence remineralization outcomes, and thus limit the translation of in vitro findings into clinical practice.^12^

### Caries type and specimen dimension

The studies investigated various carious models, including white spot lesions, artificially demineralized enamel, and dentine caries. The dimensions of specimens varied across studies, and some used sectioned premolars or molars.^10^^,^^15^ The use of an artificial caries model allows controlled lesion formation, mimicking early-stage demineralization. However, variations in lesion depth, demineralization time, and acid exposure protocols across studies introduced inconsistencies that may have affected comparability.^14^

### Sample size and grouping

A major limitation across studies was the lack of sample size justification and none provided prior power calculations, raising concerns regarding statistical robustness. The studies employed sample sizes that ranged between n = 15–40 per group, divided into treatment and control groups. These groupings enabled comparative analysis but the absence of a predefined sample size calculation weakened the reliability of the reported results, and may have led to underpowered or overestimated estimates. Future studies should incorporate sample size estimation based on statistical power analyses to strengthen the quality of the evidence obtained.

### Intervention and control groups

Bioactive glass-containing dentifrices were tested in six studies^11^^,^^12^^,^^14^, ^15^, ^16^^,^^33^ and fluoride-based pastes in five studies.^12^^,^^15^^,^^16^^,^^21^^,^^34^ These studies either used fluoride dentifrices as an intervention or control group to compare with bioactive glass and CPP-ACP formulations. In three studies,^8^^,^^14^^,^^34^ CPP-ACP formulations were tested as remineralizing agents. Studies that compared fluoride-only treatments with bioactive glass formulations consistently reported superior remineralization outcomes in bioactive glass groups.^12^^,^^14^ However, differences in treatment application protocols (e.g., frequency and duration of exposure) may have influenced the outcomes. The control groups varied across studies, including untreated enamel, placebo treatment, or fluoride-only interventions, making direct comparisons challenging.^15^

### Measured outcomes and equipment used

Standardized techniques were employed to assess remineralization effects, such as surface microhardness tests (three used Vickers^14^^,^^15^^,^^21^ and one Knoop^8^), transverse microradiography (TMR) in two studies,^11^^,^^16^ scanning electron microscopy (SEM) in four studies,^11^^,^^14^^,^^15^^,^^34^ and energy dispersive X-ray spectroscopy (EDS) in two studies.^11^ Almost all studies (81.8 %) employed microhardness for mineral recovery testing, and SEM, TMR, and microhardness testing were used to observe structural and morphological changes.^16^ These techniques provide reliable enamel assessments, but discrepancies or errors in testing parameters and measurement intervals across studies may have introduced methodological inconsistencies.^14^

### Reported findings and comparative assessment

All studies with bioactive glass-containing dentifrices reported better remineralization effects than those with fluoride-only formulations. In particular, two studies^11^^,^^12^ found that bioactive glass-containing dentifrices enhanced remineralization more effectively than dentifrices with fluoride-only formulations, and they also resulted in greater mineral deposition, lesion depth reduction, and hydroxyapatite formation. These findings were further supported in two other studies^14^^,^^15^ that demonstrated increases in enamel hardness recovery and enhanced surface integrity with bioactive glass. These findings suggest that bioactive glass is a better method for repairing early tooth damage. The CPP-ACP formulation obtained a strong remineralization effect, reinforcing its use as an auxiliary treatment to fluoride therapy.^8^

## Discussion

According to this systematic review, bioactive glass-containing dentifrices are preferable for promoting enamel and dentine remineralization, and studies support their potential use in non-invasive caries management. Most studies found that bioactive glass formulations doped with fluoride or strontium significantly increased mineral deposition and reduced the lesion depth compared with conventional fluoride-only treatments.^8^^,^^12^ Furthermore, bioactive glass increased calcium and phosphate ion concentrations, as well as fluoride, to provide a more dynamic remineralization process, with enhanced enamel repair and tubular occlusion.^11^ These findings are consistent with the growing consensus that a greater capacity for protection against dentine caries can be achieved through using multifunctional mineralization agents rather than fluoride alone.

However, although the findings are promising, definitive therapeutic conclusions cannot be drawn because of the very different methodologies employed in individual studies. Restrictions that hinder the direct comparison of results include variations in pH cycling models, lesion induction protocols, treatment application frequencies, and control groups.^14^^,^^15^ In addition, differences in specimen types, such as human versus bovine enamel, may have influenced remineralization outcomes due to differences in the composition and permeability of minerals.^16^ These differences highlight the importance of utilizing a standardized protocol for in vitro remineralization research to reduce the likelihood of unreliable or noncomparable findings.

The review identified a key limitation due to the lack of randomization, blinding, and sample size justification across all studies. Prior power calculations were not provided or described in any of the studies, which is a concern that may affect the statistical robustness of their findings.^21^ Specimen allocation to random samples and blinded outcome assessment prevents avoidable biases in the choice and assessment of outcomes that could lead to overestimation of the effectiveness of demineralizing agents.^14^ Future in vitro studies will yield better results by incorporating these methodological safeguards.

Microhardness testing (Vickers and Knoop), SEM, TMR, and EDS were consistently used to evaluate mineral recovery and structural integrity.^8^^,^^12^ These techniques provide quantitative and qualitative assessments of remineralization, and thus a better understanding of enamel and dentine repair mechanisms. Unfortunately, differences in measurement intervals and evaluation criteria made comparisons among studies difficult. Therefore, it is necessary to standardize assessment methodologies, including uniform lesion preparation, remineralization exposure times, and post-treatment evaluation criteria, to enhance the reproducibility and clinical clarity of in vitro findings.^14^

The findings considered in this review support the use of bioactive remineralization agents to advance caries prevention and non-invasive dental treatments. Despite strong in vitro evidence supporting the efficacy of bioactive glass and CPP-ACP formulations, their clinical application remains unclear due to the limitations discussed above. Further investigation is also needed because no significant difference was reported between bioactive glass and fluoride.^16^ Future research should focus on real-world settings through high-quality in situ and randomized controlled trials with standardized methodologies to allow their validation.^15^ Addressing the current methodological gaps can allow bioactive remineralization research to provide strong evidence to support the development of effective, evidence-based preventive strategies for dentine caries management.

## Conclusion

This review indicates the effectiveness of bioactive glass dentifrice, CPP-ACP, and fluoride formulations in promoting dentine remineralization, with significant improvements in mineral deposition, surface microhardness, and lesion depth reduction, emphasizing the potential use of these agents in non-invasive caries management. All studies demonstrated remineralization potential, but future research should incorporate randomization and blinding to further improve the reliability of the findings. Overall, this systematic review provides strong evidence that bioactive remineralization agents exhibit encouraging performance in vitro, but their translation into evidence-based dental applications requires stronger methodologies and validation through high-quality in situ and clinical trials.

## Ethical approval

Not applicable.

## Author contributions

Conceptualization: FTZ. Data Curation: FTZ. Investigation: FTZ and MA. Writing-Original Draft: FTZ and MA. Writing, Review, and Editing: FTZ, MA, MSZ. Approval of final Manuscript: all authors. All authors have critically reviewed and approved the final draft and are responsible for the content and similarity index of the manuscript.

## Data availability statement

Not applicable.

## Source of funding

This study was not supported by any sponsor or funder.

## Conflict of interest

All authors have no conflicts of interest to declare.
